# Draft genome sequences of two *Bifidobacterium* sp. from the honey bee (*Apis mellifera*)

**DOI:** 10.1186/1757-4749-5-42

**Published:** 2013-12-18

**Authors:** Kirk E Anderson, Andreas Johansson, Tim H Sheehan, Brendon M Mott, Vanessa Corby-Harris, Laurel Johnstone, Ryan Sprissler, William Fitz

**Affiliations:** 1 Carl Hayden Bee Research Center, USDA-ARS, Tucson AZ 85719, USA; 2 Department of Entomology, University of Arizona, Tucson AZ 85721, USA; 3 National Oceanography Centre, University of Southampton, Southampton, SO14 3ZH, UK; 4 Department of Microbiology, University of Arizona, Tucson AZ 85721, USA; 5 Bio5 Institute, University of Arizona Genomics Core, Tucson AZ 85721, USA

**Keywords:** *Bifidobacterium*, Probioiotic, *Apis mellifera*, Honey bee, Crop, Respiratory metabolic pathway, ROS tolerance

## Abstract

**Background:**

Widely considered probiotic organisms, *Bifidobacteria* are common inhabitants of the alimentary tract of animals including insects. *Bifidobacteria* identified from the honey bee are found in larval guts and throughout the alimentary tract, but attain their greatest abundance in the adult hind gut. To further understand the role of *Bifidobacteria* in honey bees, we sequenced two strains of *Bifidobacterium* cultured from different alimentary tract environments and life stages.

**Results:**

Reflecting an oxygen-rich niche, both strains possessed catalase, peroxidase, superoxide-dismutase and respiratory chain enzymes indicative of oxidative metabolism. The strains show markedly different carbohydrate processing capabilities, with one possessing auxiliary and key enzymes of the Entner-Doudoroff pathway.

**Conclusions:**

As a result of long term co-evolution, honey bee associated *Bifidobacterium* may harbor considerable strain diversity reflecting adaptation to a variety of different honey bee microenvironments and hive-mediated vertical transmission between generations.

## Background

*Bifidobacterium* are common animal commensals, used as probiotics, and widely considered important to host metabolism [1]. Most are strict anaerobes, but *Bifidobacterium asteroides* PRL22011, isolated from the honey bee hindgut, was recently sequenced and found to carry genes for oxidative respiration and protection from reactive oxygen species [2]. Moreover, a phylogenomic analysis from the same study suggests that *Bifidobacteria* associated with the honey bee is of ancient origin relative to *Bifidobacteria* in mammals. Culture based results and 454 amplicon sequencing demonstrate that *Bifdobacteria* can be found throughout the alimentary tract but reside primarily in the hind gut of honey bees [3-5]. To more thoroughly characterize the breadth of strain diversity and metabolic potential in honey bee *Bifidobacterium*, we sequenced two additional strains sampled from different honey bee alimentary tract microenvironments.

The honey bee hive is composed of a variety of nutrient rich microenvironments generated by exposed, typically continuous larval rearing and substantial food storage. These dynamic and highly variable niches support microbial communities specific to the hive environment, and are governed by a variety of biotic and abiotic factors including pH, acidity, oxygen exposure, hygroscopy, and honey bee secreted enzymes [6,7]. Following the transition of the honey bee from the larval to adult stage, the transmission of *Bifidobacteria* and other core bacteria to the gut of the newly emerged adult is seemingly accomplished via the hive environment and/or trophallaxis with older siblings [3,4]. Both of these routes expose *Bifidobacteria* to extremes of pH and oxygen found in the foregut and hive environments.

## Methods

### Bacterial culture

*Bifidobacterium* strain A11 was isolated from the gut of a third instar larvae sampled from a feral Africanized honey bee colony near Oracle, AZ [7]. Strain 7101 was isolated from the foregut (crop) of an adult nurse worker bee sampled from a managed European colony at the Carl Hayden Bee Research Center in Tucson, AZ [3]. Bacterial strains were isolated using De Man Rosaga Sharp (MRS) media under aerobic (strain A11) or microaerophilic (strain 7101) conditions at 35°C. Bacterial isolates were picked and regrown in liquid MRS media to attain enough DNA for sequencing.

### Nucleic acid isolation

A 300 μl aliquot of each MRS culture sample was centrifuged at 12,000 g for 5 min. After decanting supernatant, bacterial pellets were lyzed at 37°C for 1 h with 300 μL of lysozyme lysis buffer (100 mM NaCl, 500 mM Tris [pH 8.0], lysozyme 10 mg/ml). We then added 200 μl of SDS lysis buffer (100 mM NaCl, 500 mM Tris [pH 8.0], 10% [wt./vol.] SDS) and vortexed. After incubation at 65°C for 10 min, the mixture was centrifuged at 12,000 g for 5 min. The supernatant was transferred to another microcentrifuge tube. Protein was removed by adding 500 μl of chloroform/isoamyl alcohol (24:1), vortexing for 5 s, incubating at 4°C for 5 min, and centrifuging at 12,000 g for 5 min. The upper solution was precipitated by adding a 0.5 vol. of 7.5 M ammonium acetate and a 1.0 vol. of isopropanol. After incubation at -20°C for 15 min, DNA was pelleted at 12,000 g for 10 min and washed three times with 75% ethanol. DNA pellets were air dried, then resuspended in 100 μl of 10 mM Tris, pH 8.0.

### Library preparation and sequencing

We quantified DNA using PicoGreen, nebulized 600 ng of each sample and prepared the libraries according to the Rapid Library Preparation protocol, using Multiplex Identifiers RLMID8 and RLMID10 for strain A11 and 7101 respectively. Genome sequences were obtained at the University of Arizona Genomics Core using Roche 454 GS pyrosequencing and a whole genome shotgun strategy.

### Read quality assessment

Sequencing reads were assembled *de novo* using Roche 454 software, Newbler version 2.6 with default settings (Table 1). We used the RAST server [8] and accompanying SEED database for gene prediction and annotation (Table 2). Genome sequence submission to NCBI resulted in the reannotation of the assemblies according to the standards of the Prokaryotic Genome Automatic Annotation Pipeline (PGAAP).

**Table 1:** **Metrics associated with sequencing and assembly of two strains of *Bifidobacteria***

**Strain**	**Total reads**	**Total bases**	**Total contigs**	**N50 contig size**	**Total contig length**	**Genome coverage**	**%GC content**
7101	262,222	60,733,017	19	524,826	2,117,598	24X	59.74
A11	286,838	70,227,442	51	223,528	2,180,865	27X	60.10

**Table 2:** **Categories of functional roles (subsystems) of *Bifidobacterium *** **strains 7101 and A11 based on RAST subsystem annotation**

**Subsystem features**	**Subsystem feature counts by strain**	
	**7101**	**A11**
Cofactors, vitamins, prosthetic groups, pigments	71	70
Cell wall and capsule	42	53
Virulence, disease and defense	12	11
Potassium metabolism	2	2
Photosynthesis	0	0
Miscellaneous	6	6
Phages, prophages, transposable elements, plasmids	0	2
Membrane transport	28	12
Iron acquisition and metabolism	0	0
RNA metabolism	59	56
Nucleosides and nucleotides	61	59
Protein metabolism	157	158
Cell division and cell cycle	19	19
Motility and chemotaxis	0	4
Regulation and cell signaling	26	24
Secondary metabolism	0	0
DNA metabolism	60	62
Regulons	0	0
Fatty Acids, lipids, and isoprenoids	31	29
Nitrogen metabolism	7	7
Dormancy and sporulation	1	1
Respiration	21	21
Stress response	41	41
Metabolism of aromatic compounds	3	3
Amino acids and derivatives	155	158
Sulfur metabolism	13	12
Phosphorus metabolism	22	22
Carbohydrates	183	235

## Quality assurance

Throughout many steps of the process, Sanger sequencing of the 16S rRNA gene confirmed that both isolates were pure and >99% similar to previously submitted *Bifidobacterium* sequences. *B. asteroides* PRL2011 differed from each strain at 10 of 1473 16S rDNA nucleotide positions. Strain A11 and 7101 differed from one another at 4 of 1473 nucleotide positions.

## Initial findings

Both strains lack the glycolytic enzyme phosphofructokinase-1, but possess the enzymatic marker indicative of genus *Bifidobacterium*: fructose-6-phosphate phosphoketolase, historically referred to as the “bifid shunt” [9]. Unlike typical *Bifidobacterium*, and as described previously for honey bee associated *Bifidobacterium*[2], both strains also possess oxidative respiratory pathways, and genes that cope with reactive oxygen species, including catalase, peroxidase and superoxide-dismutase. Consistent with co-evolution in and around harsh osmoregulatory conditions [6,7], the transmembrane channel aquaporin Z was present in both genomes. This protein is highly stable, facilitates both rapid and long term osmoregulation, and resists denaturing due to heat, detergent, or extremes of pH.

Absent in strain 7101, strain A11 possesses genes for chemotaxis, and the Entner-Doudoroff pathway. Found in many pathogenic bacteria [10], strain A11 has the dTDP-rhamnose biosynthetic pathway, which may play a part in cell wall integrity, growth and/or host interaction. Lacking in strain A11, strain 7101 contains 5 different EFC class transporters dedicated to the importation of vitamins, and 4 CRISPR-associated proteins predicted to provide immunity against genetic parasites.

## Future directions

The extent of strain diversity and associated function of *Bifidobacteria* in honeybees remains unclear. Identification of the metabolic potential of different strains provides information on the predicted survival of unique strains in different gut and hive microenvironments. Comparative transcriptomics under different environmental conditions may elucidate candidate strains for probiotic treatment, a viable alternative or complement to traditional treatments typically applied to honey bee colonies.

## Availability of supporting data

The draft genome sequences of *Bifidobacterium* strain A11 and strain 7101 were deposited in DDBJ/EMBL/GenBank under the accessions AWUO00000000 and AWUN00000000 respectively.

## Competing interests

The authors declare no competing interests.

## Authors’ contributions

KEA planned the experiments and wrote the paper. AJ, BMM and THS cultured, isolated, and identified the bacteria. WF and VCH extracted the DNA and provided genome metrics. LJ and RS processed the genome assemblies. All authors read and approved the final manuscript.
